# Ultrahigh pressure-induced modification of morphology and performance of MOF-derived Cu@C electrocatalysts†

**DOI:** 10.1039/d2na00829g

**Published:** 2022-11-29

**Authors:** Ichiro Yamane, Kota Sato, Teruki Ando, Taijiro Tadokoro, Seiya Yokokura, Taro Nagahama, Yoshiki Kato, Tatsuya Takeguchi, Toshihiro Shimada

**Affiliations:** a Graduate School of Chemical Sciences and Engineering, Hokkaido University Kita 13 Nishi 8, Kita-ku Sapporo 060-8628 Japan shimadat@eng.hokudai.ac.jp; b Division of Applied Chemistry, Faculty of Engineering Hokkaido University Kita 13 Nishi 8, Kita-ku Sapporo 060-8628 Japan; c Department of Chemistry, Faculty of Science and Engineering, Iwate University 4-3-5 Ueda Morioka 020-8551 Japan

## Abstract

We report the pyrolysis of copper-containing metal–organic frameworks under high pressure and the effect of the applied pressure on the morphology and electrocatalytic performance toward the oxygen-related reactions of the products. The high-pressure and high-temperature (HPHT) syntheses were performed under 5, 2.5, 1, and 0.5 GPa, and the Cu@C products were obtained except for the 2.5 GPa experiment. Copper formed a shell-like nanostructure on the carbon matrices during the 0.5 GPa experiment, whereas copper formed sub-nanometer sized particles in the carbon matrices with the increasing pressure. It is considered that the transportation of copper atoms by outgassing during the pyrolysis affects the morphology. Electrochemical measurements revealed that all samples exhibited activity for the oxygen reduction reaction (ORR). The 0.5 GPa-treated product also exhibited the oxygen evolution reaction (OER). The overall ORR/OER performance of this product was excellent among Cu-based bifunctional materials even though it did not contain cocatalysts such as nitrogen-doped carbon or other metal elements. The Cu(iii) species in the nano-thick copper shell structure provided the active sites for the OER.

## Abstract 2

We report the pyrolysis of copper-containing metal–organic frameworks under high pressure and the effect of the applied pressure on the morphology and electrocatalytic performance toward the oxygen-related reactions of the products.

## Graphical abstract

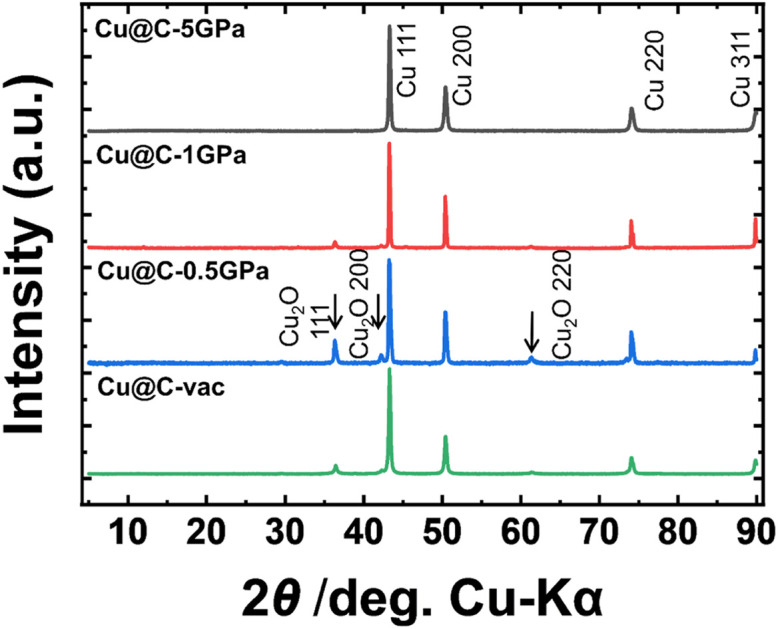

## Introduction

The development of high-performance catalysts has currently been paid attention to deal with environmental issues and promote clean and sustainable energy production.^1–3^ Although noble metals, such as Pt, Pd, and Rh, are mainly used as catalysts, their widespread use is prevented by their low earth abundance and high prices.^4–6^ In particular, electrocatalysts for the oxygen reduction reactions (ORR) and oxygen evolution reaction (OER) will significantly increase in demand because they play an important role in clean energy technologies, *i.e.*, water splitting,^7,8^ fuel cells,^9,10^ and metal–air batteries.^11^ Therefore, high-performance materials as alternatives to noble metals are strongly desired and have been studied.^12–14^ There are two approaches to develop them. One is the search for novel compounds with previously unknown compositions, such as new transition metal oxynitrides,^15–17^ Heusler alloys,^18,19^ and MXenes.^20^ The other one is controlling the nanostructures of materials to improve the catalytic efficiency. What is expected from this approach includes making their active sites more dispersed,^21^ modifying their electronic structure,^22–24^ and stabilizing specific atomic arrangements on the nanosurfaces.^25–27^ Appropriately predesigned precursors are needed in this approach. An example of the former approach includes high-pressure synthesis. High-pressure synthesis has been used to search for novel materials because this method can provide compounds difficult to synthesize under ambient conditions.^28–30^ Previous studies regarding the high-pressure synthesis of electrocatalysts were limited, which include quadruple perovskites,^31,32^ transition metal carbides,^33^ phosphides,^34^ and borides.^35^ However, no studies of the latter approach – controlling the nanostructures of electrocatalysts – have utilized high-pressure synthesis before the present authors.

We have employed metal–organic frameworks (MOFs) for controlling the nanostructures by high-pressure and high-temperature (HPHT) treatments.^36^ MOFs consist of metal cations and organic ligands, and carbon-supported heterogeneous catalysts can be synthesized in one step by pyrolysis.^37–39^ However, this approach often suffers from the aggregation of the catalyst particles during the pyrolysis. Metals that are not alloyed with carbon, ex. Cu, strongly indicate this tendency.^38,40^ Although using designed precursors may solve this problem,^41–48^ this method also has challenges. First, designing and synthesizing precursors require complicated procedures. Second, available metal elements are limited because precursor MOFs suitable for this method are restricted.^21^ We focus on the HPHT treatments of the MOFs as a new method to control the nanostructures.

Generally, the lattice diffusion of atoms is inhibited under high pressure due to an increase in the activation energy of the diffusion.^49–52^ This feature suppresses the agglomeration of metal atoms in the thermal treatments during the synthesis of the catalysts. Therefore, the HPHT treatment of the MOFs will lead to the miniaturization of nanoparticles in the MOF-derived heterogeneous catalysts. We have already succeeded in the preparation of single-nm copper particles on carbon supports (Cu@C) without nitrogen anchoring simply by the HPHT treatments of copper(ii)-benzene-1,3,5-tricarboxylate (Cu-BTC) at 5 GPa.^36^ However, the behavior under lower pressure conditions remains unknown. The electrocatalytic activity has also not been studied.

We now report the morphology of Cu for the Cu@C products synthesized by annealing Cu-BTC at various pressures and their electrocatalytic performance toward oxygen-related reactions in alkaline media. An anomalous pressure dependence was observed, and their performance was superior to that of MOF-derived catalysts in previous reports. The findings will pave the way for the application of high-pressure techniques for controlling the morphology and activity of nanostructured catalysis.

## Experimental section

### Materials and sample preparation

The Cu@C samples were synthesized from Cu-BTC pellets by HPHT treatments under various pressure conditions using a DIA-type cubic-anvil press (CT-factory, Tokyo) or thermal treatment in a vacuum-sealed glass tube. The precursor Cu-BTC was purchased from Sigma Aldrich (its trade name is Basolite® C-300). The applied pressures used in the HPHT treatment were 5, 2.5, 1, and 0.5 GPa, and the heating temperature and time were 500 °C and 15 min, respectively. The cell assembly for the HPHT experiments consisted of a pyrophyllite block with a hole, pyrophyllite disks, a graphite tube coated inside with h-BN, an h-BN pellet, stainless steel rings, and a chromel–alumel thermocouple in an Al_2_O_3_ tube (Fig. S1†). The sample and h-BN pellet were encapsulated in the graphite tube, and then the tube was placed in the hole of the pyrophyllite block. The h-BN pellet was used as a spacer. The hole in the block was closed by fitting the pyrophyllite disks and stainless steel rings. Finally, we drilled a diagonal hole through the block into which we inserted the thermocouple with the Al_2_O_3_ tube. The hole penetrated the h-BN spacer within the graphite tube to prevent damaging the sample pellets, and a temperature measuring junction was placed at the center of the graphite tube. The sample was heated by flowing an electric current to the graphite heater *via* the anvils and stainless-steel rings during the HPHT treatments. The heating started after the cell assembly was pressed to the target pressure. The thermal treatment of a vacuum-sealed Cu-BTC pellet was also conducted at 500 °C for 15 min. Subsequently, the sample was recovered from the glass tube under ambient conditions. After removing the impurity materials due to the cell assembly, each obtained sample was ground using a mortar and pestle, and then used for the characterization studies and electrochemical measurements.

### Characterization

Powder X-ray diffraction (XRD) measurements were performed using a MiniFlex-600 (Rigaku, Cu Kα (*λ* = 1.5405 Å)). Transmission electron microscopy (TEM) observations were conducted using a JEM-2010 (JEOL). High-angle annular dark-field scanning-TEM (HAADF-STEM) observations and TEM electron energy loss spectroscopy (TEM-EELS) measurements were performed using a Titan3 G2 60-300 (FEI). TEM/STEM observations and EDS measurements shown in Fig. S3–S5† were performed using a JEM-2100 (JEOL). X-ray photoelectron spectroscopy measurements were performed using a JPS-9200 (JEOL, using Mg Kα X-ray).

### Electrochemical measurements

All the electrochemical measurements were carried out in a 0.1 M KOH aqueous solution at room temperature using a rotating-disk electrode system (BAS, RRDE-3A) and potentiostat (Hokuto Denko, HSV-110). The working electrode (WE), reference electrode (RE), and counter electrode (CE) in this study were a glassy carbon (GC) rotating-disk electrode (*d* = 0.4 cm), a Hg/HgO electrode, and a Pt coil electrode, respectively (Fig. S2†).

The catalyst ink was prepared as follows: the sample powder (4 mg) was added to a mixture of a 60 μL Nafion 5 wt% solution (Sigma-Aldrich) and 540 μL of 99.5% ethanol (Japan Alcohol Trading), and then the suspension was sonicated for 30 min to yield a uniform ink. The prepared ink (8 μL) was dropped onto a GC disk WE and dried for 30 min. The potentials *vs.* a reversible hydrogen electrode (RHE) were calculated using the following formula:^10^

1 *E*_RHE_ = *E*_Hg/HgO_ + 0.098 V + 0.0591 × (pH of the electrolyte)

where *E*_RHE_ and *E*_Hg/HgO_ are the potentials *versus* RHE and Hg/HgO RE, respectively.

We analyzed the electron transfer number (*n*) for the ORR from the linear sweep voltammograms measured at various rotating-speeds using the Koutecký–Levich equation:^53^

2 *i*^−1^ = *i*_k_^−1^ + *i*_L_^−1^ = *i*_k_^−1^  + 1/(0.620 *nFcD*^2/3^*ν*^−1/6^*ω*^1/2^)

where *I*, *i*_k_, *i*_L_, *F*, *c*, *D*, *ν*, and *ω* are the current density measured at the disk electrode, the kinetic-limited current density, the mass-transfer-limiting current density, Faraday's constant, the oxygen concentration of O_2_ in the electrolyte, the diffusion coefficient of the O_2_ in the electrolyte, the kinematic viscosity of the electrolyte, and the angular velocity of the disk electrode, respectively.

## Results & discussion

### Sample preparation

Gas emissions and blowouts to the outside were not observed during the HPHT experiments at 5 GPa, whereas gentle gas emissions to the outside and intense blowouts of the sample cell assembly occurred at 0.5 and 2.5 GPa, respectively. The emissions were observed by temporary drops in the applied loads and sounds. The blowouts destroyed the cell assembly and anvils, which prevented conducting a further analysis at 2.5 GPa. We recovered the sample pellets from the press equipment under ambient conditions after the experiment at 5, 1, and 0.5 GPa. These recovered products from the experiments at 5, 1, and 0.5 GPa were labeled as Cu@C-5GPa, Cu@C-1GPa, and Cu@C-0.5GPa, respectively. We also obtained the products after the thermal treatments in the vacuum-sealed glass and labeled the products as Cu@C-vac.

### Structural characterization of the samples

**Fig. 1:**
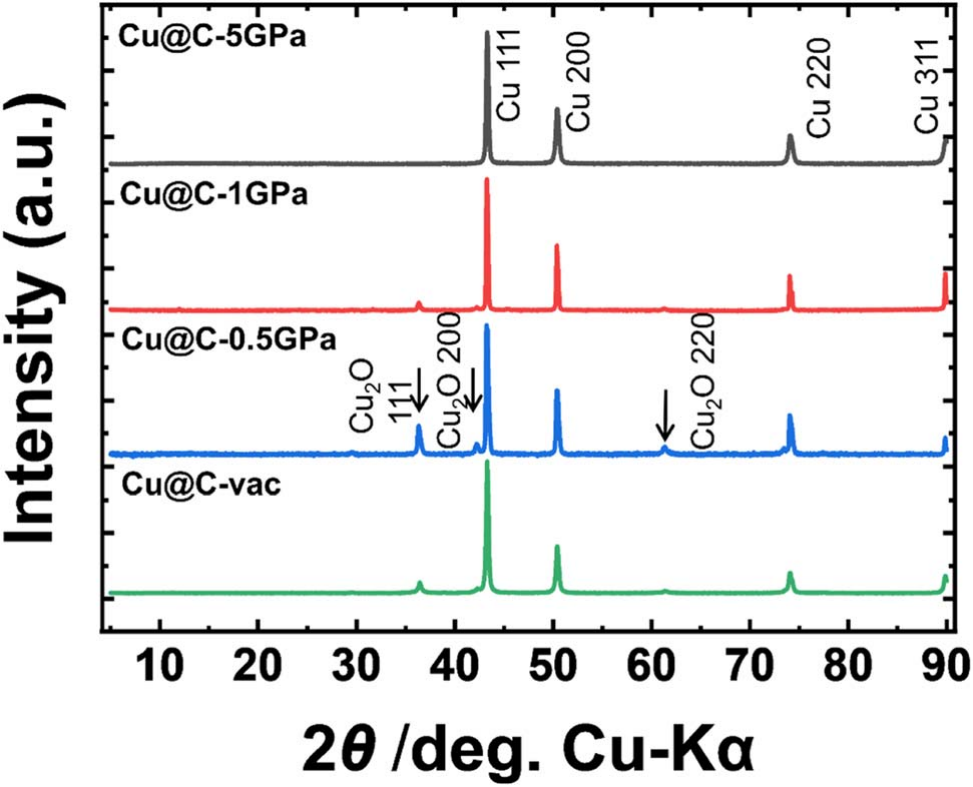
Powder XRD patterns of the samples pyrolyzed at various pressures.

We carried out a powder X-ray diffraction (XRD) measurement of the samples recovered after the treatments to identify the decomposition products. Fig. 1 shows the XRD profiles of Cu-BTC pyrolyzed under high pressure or in the vacuum-sealed glass tube. All the profiles exhibited obvious diffraction peaks at 43.3°, 50.4°, 74.0°, and 89.8°. These are attributed to the (111), (200), (220), and (311) planes of copper, respectively. This result indicated that most of the Cu^2+^ cations in Cu-BTC did not form oxides but were reduced to Cu^0^ during the treatments. This reduction reaction is likely to have been caused by decarboxylation of the precursor. Small diffraction peaks due to Cu_2_O were also observed at 36.3°, 42.2°, and 63.3° in the patterns of Cu@C-1GPa, Cu@C-0.5GPa, and Cu@C-vac, whereas no Cu_2_O peaks were observed in the pattern of Cu@C-5GPa. The relative intensities of the Cu_2_O diffraction peaks decreased as the applied pressures increased in the HPHT experiments. This suggested that the precursor was less exposed to the air when it was pyrolyzed at high pressure. However, a characteristic peak at around ∼26° derived from graphitic carbon was not observed in all the samples. This indicated that the organic ligands of Cu-BTC were not graphitized during the carbonization. In summary, the Cu^2+^ cation and organic ligands turned into metallic Cu or Cu_2_O and carbon matrices with a low graphitization degree, respectively.

Previous studies regarding the pyrolysis of Cu-BTC at ambient pressure are summarized in Table S1.† The XRD results from those studies included only the peaks of Cu or Cu_2_O from the as-prepared samples. The XRD peak of graphitic carbon appeared only after removing the copper by acid washing, which suggests that the graphite peak was very weak. This is consistent with the absence of a graphite peak in the present study. Table S1† also reveals that a high temperature and long heating time, for example, 800 °C for 2 h, were required to obtain only metallic Cu from the pyrolysis in an inert atmosphere. However, we successfully obtained the product without oxides from the 5 GPa thermal treatment at a much lower temperature for a shorter time, that is, 500 °C for 15 min, despite being performed in air. These results indicated that the high-pressure treatment can suppress the formation of copper oxides.

**Fig. 2:**
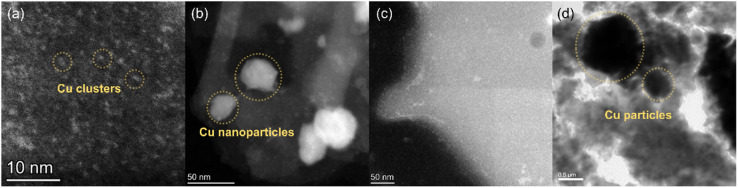
HAADF-STEM images of (a) Cu@C-5GPa, (b) Cu@C-1GPa, and (c) Cu@C-0.5GPa, and the TEM image of (d) Cu@C-vac.

The morphology of the Cu species in the product was investigated by TEM and STEM (Fig. 2). HAADF-STEM observations revealed that nanoclusters and nanoparticles supported on the matrices existed in Cu@C-5GPa and Cu@C-1GPa, respectively (Fig. 2a and b). The materials of these nanoparticles and the matrices were identified as copper and carbon, respectively, using the HAADF-STEM contrast and EDS analysis. This assignment was confirmed by TEM/STEM-EDS point analysis and mapping measurements (Fig. S3–S5†). The result proved that the products had copper supported on carbon (Cu@C) nanostructures. The sizes of the nanoparticles in Cu@C-5GPa and Cu@C-1GPa had diameters of ∼1 nm and tens of nm, respectively, whereas Cu@C-vac contained large particles with a diameter of >1 μm (Fig. 2d). The sizes of the copper particles in the products are controlled by the applied pressure during the pyrolysis, indicating that the ultrahigh pressure effectively modulated the atomic diffusion.

**Fig. 3:**
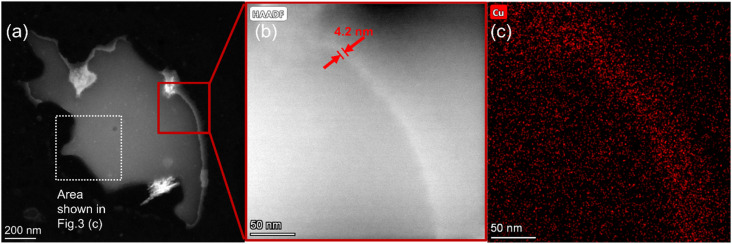
HAADF-STEM images at (a) low magnification and (b) and (c) HAADF-STEM images at high magnification of Cu@C-0.5GPa and the corresponding EDS mapping of Cu.

For Cu@C-0.5GPa, however, copper did not form particles but was uniformly distributed over the sample powder (Fig. 2c). Fig. 3a and b show that the edge of the powder contained copper at a high concentration, which was confirmed by the EDS mapping as shown in Fig. 3c. Thus, it was found that the morphology of copper in this sample was different from that in the other ones and formed thin shells with a thickness of ∼4 nm on the surface of the carbon matrices.

**Fig. 4:**
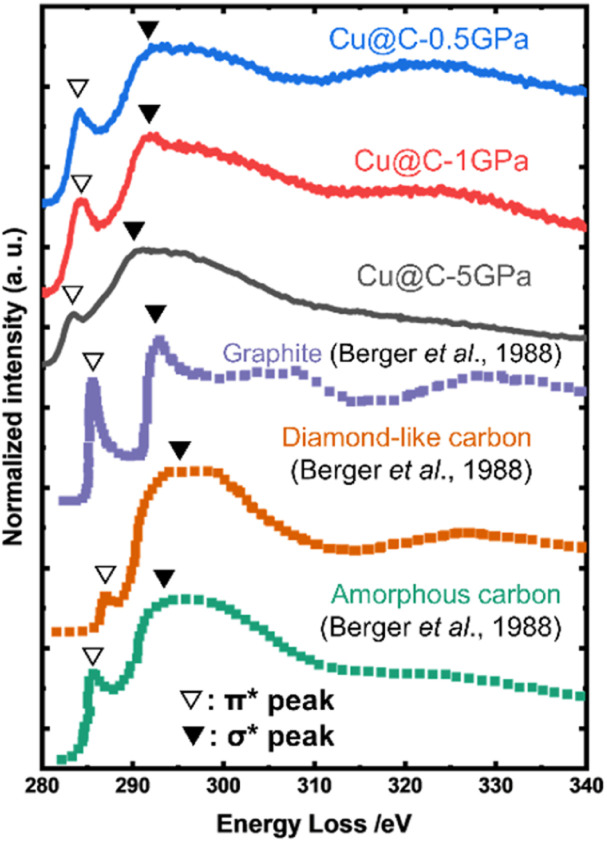
TEM-EELS spectra near the carbon K-edge of the HPHT-treated samples and reference substances. ∇ and ▼ are the π* and σ* peaks, respectively. The spectra of the graphite, diamond-like carbon, and amorphous carbon were reproduced from the data of Berger *et al.*^54^

To further evaluate the carbon matrices of Cu@C in the HPHT-treated products, the core-loss EELS spectra of the carbon K-edge were obtained using TEM. Fig. 4 shows the EELS spectra of our products and those of reference materials traced from ref. 54. The spectra of our products had two peaks near 285 and 291 eV the same as those of the references, and their shapes were similar to that of amorphous carbon rather than graphite. These two peaks are called π* and σ* peaks, which are derived from the π and σ bonds, respectively.^54,55^ We estimated the graphitization degree of the sample from the area ratios of the π* peak to the K-edge. The area of the π* peak was calculated by peak separation using the Gaussian function in the range of 280–295 eV. The area of the K-edge was defined as the area in the range of 280–310 eV. The baseline in the above calculations was set to the intensity at 280 eV. As a result, the π* peak/K-edge ratios normalized by that of graphite were 56% for Cu@C-0.5GPa, 76% for Cu@C-1GPa, 23% for Cu@C-5GPa, 17% for the diamond-like carbon, and 49% for the amorphous carbon. The EELS results revealed that the carbon matrices of the products did not graphitize and formed amorphous carbon. In particular, Cu@C-5GPa had a remarkably poor graphitization degree among the products. Cu nanoparticles in the matrix of Cu@C-5GPa were much smaller than those in the other products (Fig. 2) and these fine particles probably produced more defects in the matrix. This highly defective structure probably results in a remarkably poor graphitization degree.

The drastic morphology change in Cu@C-0.5GPa seems to be related to the outgassing during the HPHT treatments. Cu-BTC is a kind of carboxylate salt, which leads to CO_2_ evolution by decomposition of the carboxylate groups during heating Cu-BTC. For the experiment at 5 GPa, the evolved CO_2_ was confined inside the cell assembly by the ultrahigh applied pressure and it could not go out of the cell. On the other hand, the gentle gas emission to the outside during the treatment was observed at 0.5 GPa. We consider that Cu atoms are carried by CO_2_ gas or its gaseous precursors (small Cu-complexes) to the grain boundary of the MOF and deposited there. When the sample is retrieved to ambient conditions, the grains are separated at the grain boundary. As a result, the thin copper shell is exposed at the surface of the particles.

We measured the XPS spectra of the samples to evaluate the Cu valency in detail. Fig. S6a† shows the XPS narrow scan spectra of Cu 2p_3/2_. The broad Cu^2+^ satellite peaks were observed clearly in Cu@C-5GPa and Cu@C-1GPa, whereas those in Cu@C-0.5GPa and Cu@C-vac were weak. The result after peak deconvolution is summarized in Table S2.† We also measured Cu LMM AES spectra to distinguish Cu^0^ and Cu_2_O (Fig. S6b†) because they show almost the same chemical shift in Cu 2p_3/2_ XPS spectra. AES showed Cu_2_O and Cu^0^ peaks at ∼916.8 eV and ∼918.6 eV, respectively. It is noted that Cu@C-5GPa did not show the Cu^0^ component while others did. These results provide the following insight about Cu valences: the average valences of the surface Cu decrease in the order of Cu@C-5GPa, Cu@C-1GPa, Cu@C-0.5GPa, and Cu@C-vac. This suggests that the surface of Cu nanoparticles is easily oxidized in the air.

### Electrocatalytic ORR performance

**Fig. 5:**
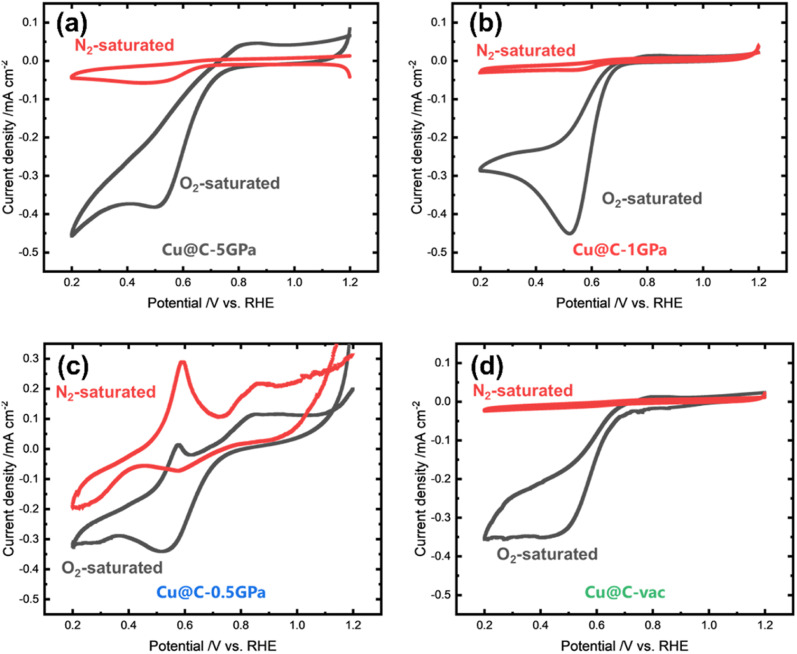
CV curves of (a) Cu@C-vac, (b) Cu@C-5GPa, (c) Cu@C-1GPa, and (d) Cu@C-0.5GPa at 10 mV s^−1^ scan rate. The red and black curves are measured in N_2_-saturated and O_2_-saturated 0.1 M KOH aqueous solutions, respectively.

Cyclic voltammetry (CV) curves in a N_2_ or O_2_ saturated 0.1 M KOH aqueous solution were measured to test the electrocatalytic activities toward the ORR. As shown in Fig. 5, each CV curve, except that of Cu@C-0.5GPa, exhibited a cathodic peak in only the O_2_-saturated solutions. It means that the reaction attributed to the cathodic peak was the reduction reaction related to O_2_. The shapes of the CV curves resemble that of the ORR in the irreversible, electron-transfer-limited case.^56–60^

The CV curve of Cu@C-0.5GPa showed different features from others. Namely, its shape was different from those of the other products and had not only cathodic but also anodic peaks. The anodic peaks are not derived from the ORR-related reactions, but from the oxidation reactions of copper (2Cu + 2OH^−^ → Cu_2_O + H_2_O + 2e^−^) because they were even observed in the N_2_-saturated electrolyte. For comparison, the CV curves of bulk Cu and the Cu monolayer on Pt in an Ar-saturated alkaline medium are shown in Fig. S7† along with those of Cu@C-0.5GPa (the CV data of bulk and monolayer Cu were reproduced from Giri *et al.*^61^). As shown in Fig. S7,† bulk copper was initially oxidized to Cu_2_O at 0.6 V *vs.* RHE. Subsequently, Cu_2_O was further oxidized to Cu(OH)_2_ and CuO *via* Cu(OH)_4_^2−^ in two steps at 0.9–1.1 V *vs.* RHE. The Cu monolayer was also reported to show some oxidation peaks. However, the peak positions were shifted to positive compared to that of bulk copper and the loop shape was different from that of the bulk. This is commonly observed in thin film heterostructures, for example, Giri *et al.*^61^ explained that the peak shift was caused by the Pt underneath the Cu monolayer. Pt gave nobility to Cu because of the good affinity of Cu for Pt.

Compared to the Cu bulk and monolayer (Fig. S7†), the CV curve of Cu@C-0.5GPa had oxidation peaks at the same positions as the bulk CV curve. Namely, the anodic peaks observed at 0.6 and 0.9–1.1 V were assigned to the oxidation from metallic Cu to Cu_2_O and the further oxidation to CuO and Cu(OH)_2_. In contrast, the shape of the CV curve of Cu@C-0.5GPa was similar to that of the Cu monolayer. These discrepancies can be explained by assuming that the shape of the CV curve is related to the morphology of copper. As shown in Fig. 3, the copper in Cu@C-0.5GPa was a thin layer (∼4 nm-thick), not the bulk. We consider that this Cu layer has microstructures and its electronic structure is similar to that of a monolayer, thus affecting the CV characteristics.

CV curves of Cu@C-0.5GPa had cathodic peaks with different potentials in the N_2_ and O_2_-saturated electrolytes. The peak observed in the N_2_-saturated electrolyte corresponded to the reduction of CuO to Cu. The intensity of the peak was weaker than that of the anodic peak at 0.6 V *vs.* RHE because the copper atoms of the electrode presumably dissolved in the electrolyte as Cu(OH)_4_^2−^. On the other hand, in the O_2_-saturated electrolyte, the cathodic peak was enhanced and shifted to the negative side. This shows that this cathodic peak was derived from the ORR as well as the CuO reduction reaction. In summary, Cu@C-0.5GPa showed CV curves with unique shapes because the copper atoms formed thin layers. The thin copper layers had a reactivity for oxidation while other products did not.

**Fig. 6:**
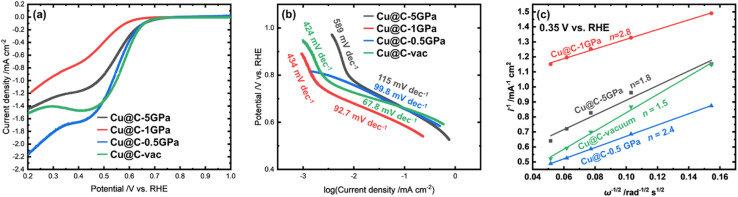
(a) LSV curves of the ORR for the products in O_2_-saturated 0.1 M KOH aqueous solution at rotation rates of 1600 rpm and scan rates of 5 mV s^−1^. (b) Tafel plot of the ORR for the products. (c) Koutecký–Levich plots of the products at 0.35 V *vs.* RHE, where *i* and *ω* are disk current density and disk rotating speed, respectively.

Fig. 6a shows the linear sweep voltammetry (LSV) results in the O_2_-saturated 0.1 M KOH aqueous solution using a rotating disk electrode (RDE) for detailed evaluations of the ORR activity. The onset potentials, *E*_onset_, of the samples synthesized at Cu@C-5GPa, Cu@C-1GPa, Cu@C-0.5GPa, and Cu@C-vac were 0.66, 0.61, 0.65, and 0.66 V *vs.* RHE, respectively, implying that the HPHT-treatments did not improve the *E*_onset_. Each LSV curve had kinetic-, mixed- and diffusion-controlled regions though the plateau in the diffusion-controlled region was not very clear. This suggests the poorer electronic conductivity of the sample due to the low graphitization degree.

Tafel plots from the LSV curves measured at 1600 rpm are shown in Fig. 6b to evaluate the kinetics of the ORR in detail. The samples, except for Cu@C-0.5GPa, had two steps corresponding to different Tafel slopes, indicating that the ORR mechanism changes with the potential. This phenomenon can be caused by a decrease in the coverage of the absorbed species on the catalyst or the oxidation number on the active sites.^62,63^ The analysis of the electron transfer numbers regarding the ORR was conducted using the Koutecký–Levich plots. As shown in Fig. 6c, the plots demonstrate good linearity. The electron transfer numbers *n* of Cu@C-5GPa, Cu@C-1GPa, Cu@C-0.5GPa, and Cu@C-vac were calculated from the slope of each plot, resulting in about 1.8, 2.8, 1.5, and 2.4, respectively. This indicated that the ORR for each product dominantly proceeds not by the four-electron pathway that generates H_2_O, but by the two-electron pathway that generates hydrogen peroxide. We did not observe any significant synergetic role of Cu_2_O in Cu@C-0.5GPa in the ORR data.

### Electrocatalytic OER performance

**Fig. 7:**
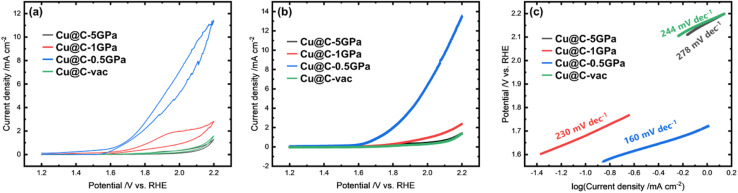
(a) CV curves of the OER for the products in N_2_-saturated 0.1 M KOH aqueous solution at a scan rate of 10 mV s^−1^ and (b) LSV curves of the OER for the products in N_2_-saturated 0.1 M KOH aqueous solution at a rotation rate of 1600 rpm and scan rate of 5 mV s^−1^. (c) Tafel plot of the OER for the products.

We conducted CV measurements in N_2_-saturated 0.1 M KOH aqueous solutions to evaluate the OER electrocatalytic activities of the products (Fig. 7a). The CV curve of Cu@C-0.5GPa showed distinct catalytic activity toward the OER. In addition, the curve exhibited a cathodic peak at 1.5–1.6 V *vs.* RHE. This cathodic peak had been assigned to the reduction of Cu(iii) to Cu(ii) in previous studies.^64–66^ This indicated that Cu@C-0.5GPa include Cu(iii) species during the oxidation scan. The kinetics of the OER in each product was evaluated by the LSV measurements at rotation scans of 1600 rpm (Fig. 7b). The Tafel plots obtained by the LSV curves are shown in Fig. 7c. The Tafel slopes of Cu@C-5GPa, Cu@C-1GPa, Cu@C-0.5GPa, and Cu@C-vac were 278, 230, 160, and 244 mV dec^−1^, respectively. These results revealed that CU@C-0.5GPa works as a bifunctional electrocatalyst for the OER/ORR.

The CV curve of Cu@C-0.5GPa in Fig. 7a indicated that Cu(iii) species was present in Cu@C-0.5GPa during the oxidation scan for the OER. This result is supported by the fact that Cu was oxidized to the Cu(ii) state in the scan from 0.2 to 1.2 V *vs.* RHE (Fig. 5 and S7†). It was reported that Cu(iii) species enhances the OER catalytic activity,^64,67^ and this probably produces the highest OER performance for Cu@C-0.5GPa. On the other hand, the copper in other products exhibited poor activity for the OER.

In those products, copper formed a morphology different from that of Cu@C-0.5GPa, with fine particles embedded in the carbon matrices. Cu@C-5GPa and Cu@C-1GPa contained Cu NPs with an oxidated surface (Fig. S6†), and the CV results indicate that the surface had low activity. Cu@C-vac included the Cu particles with larger size and less oxidated surface than the others. The CV results indicated that the particles exhibited an oxidation resistance. It suggests that the surface of the particles is encapsulated by a very thin shell of amorphous carbon. In summary, the difference in the morphology would affect the oxidation state of copper during the electrochemical processes, and then these states modified the OER performance.

### The comparison with other copper-based bifunctional electrocatalysts for the ORR/OER

**Fig. 8:**
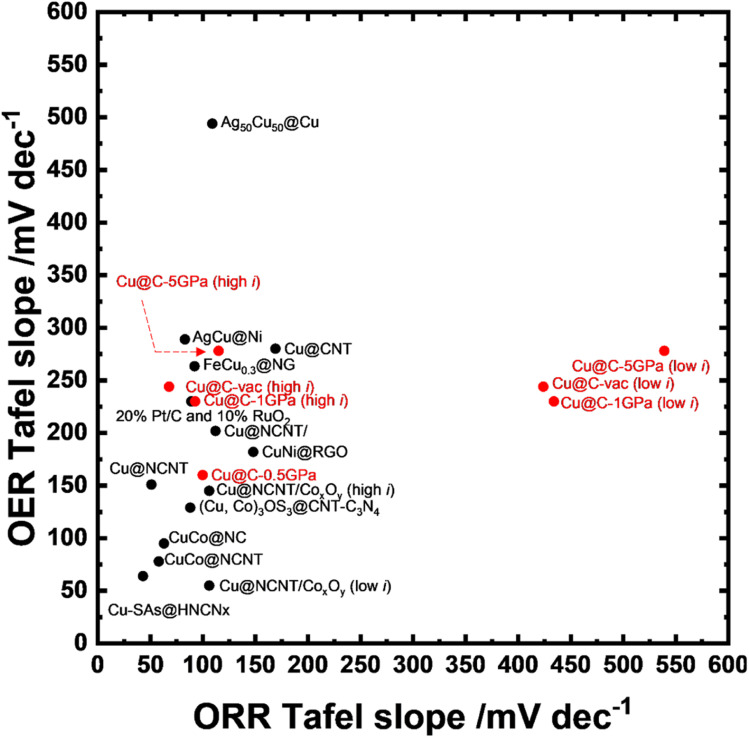
The performance comparison of the reported copper-based bifunctional electrocatalyst. The plotted points were the data from the contents of Table S3.† The red points are the data from this study.

The performance comparison of recently-reported Cu-based ORR/OER bifunctional electrocatalysts is shown in Fig. 8 and Table S3.† In Fig. 8, when the distance between a plotted point and the origin is smaller, the performance of the electrocatalyst is higher. This is because the lower value of the Tafel slopes means faster kinetics in the electrochemical processes. Thus Fig. 8 reveals that the overall performance of Cu@C-0.5GPa was superior to that of most of the copper-based OER/ORR bifunctional electrocatalysts except for the Co-containing ones. As shown in Table S3† and Fig. 8, the Cu-based OER/ORR electrocatalysts are often supported on nitrogen-doped carbon (NC) for enhanced performance. NC is useful for the following reasons: the pyridinic-N in NC itself acts as the electrocatalyst for the ORR;^68^ it can anchor metal atoms with N atoms that improved their dispersion.^21^Fig. 8 also shows that the Cu-based OER/ORR electrocatalysts were often combined with other metal elements, such as Co, Ni, and Fe, to improve their performance. It is noted that Cu@C-0.5GPa exhibited an overall performance comparable to or higher than those even though it does not contain NC or other metal elements. This result revealed that the performance of Cu-based OER/ORR bifunctional electrocatalysts can be further improved by controlling the morphology of copper nanostructures.

## Conclusions

We synthesized Cu@C composite materials from Cu-BTC by high-pressure and high-temperature treatments, where the applied pressures were 5, 1, and 0.5 GPa. Copper formed fine particles in the carbon matrices in the experiments at 5 and 1 GPa and the sizes became smaller with an increase in the applied pressure. On the other hand, copper formed thin layers with thicknesses of ∼4 nm on the carbon matrices at 0.5 GPa. The nano-layered Cu morphology at 0.5 GPa probably comes from the transportation and the deposition of Cu atoms at grain boundaries during the CO_2_ evolution from the decomposed MOF. The difference in the oxidation resistance affected the electrocatalytic performance, and the OER performance of the 0.5 GPa-treated product was enhanced by the Cu(iii) species generated during the oxidation scan. The 0.5 GPa-treated product can work as a bifunctional electrocatalyst because it also exhibited activity for the ORR as with other products. Its overall performance was comparable to that of other copper-based bifunctional electrocatalysts which contain N-doped carbon or other metals such as Ni and Fe. A pressure of 0.5 GPa can be generated by large-volume high-pressure apparatuses such as piston cylinders. Therefore, the method proposed in this study is feasible for mass production.

## Conflicts of interest

There are no conflicts to declare.

## Supplementary Material

NA-005-D2NA00829G-s001
