# Supplementary material for: Ultrahigh pressure-induced modification of morphology and performance of MOF-derived Cu@C electrocatalysts
Source: Nanoscale Adv. 2022 Nov 29;5(2):493–502. doi: 10.1039/d2na00829g (PMC9846442; doi:10.1039/d2na00829g)
Supplement: NA-005-D2NA00829G-s001 [file NA-005-D2NA00829G-s001.pdf]

Electronic Supplementary Material (ESI) for Nanoscale Advances.
This journal is © The Royal Society of Chemistry 2022

Supporting Information

Title: Ultrahigh pressure-induced modification of morphology and performance of MOFs-derived Cu@C electrocatalysts.

Authors: Ichiro Yamane[a], Kota Sato[a], Teruki Ando[b], Taijiro Tadokoro[b], Seiya Yokokura[a,b], Taro Nagahama[a,b], Yoshiki Kato[c] , Tatsuya Takeguchi[c], Toshihiro Shimada[a,b].

[a] Graduate School of Chemical Sciences and Engineering, Hokkaido University, Kita 13 Nishi 8, Kita-ku, Sapporo, 060-8628, Japan

[b] Division of Applied Chemistry, Faculty of Engineering Hokkaido University, Kita 13 Nishi 8, Kita-ku, Sapporo, 060-8628, Japan

[c] Department of Chemistry, Faculty of Science and Engineering, Iwate University, 4-3-5 Ueda, Morioka 020-8551, Japan

1. Details of apparatus

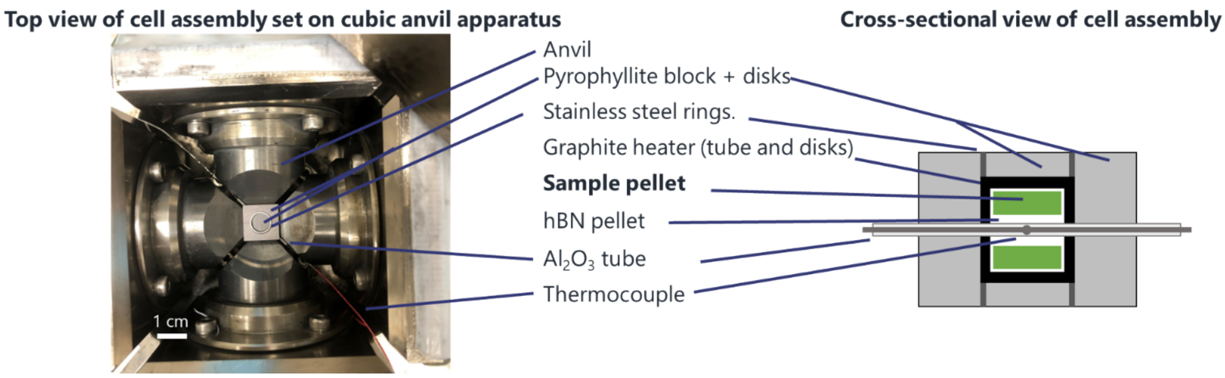

Fig. S1 The schematic image of the cross-sectional view of the cell assembly for HPHT experiments.

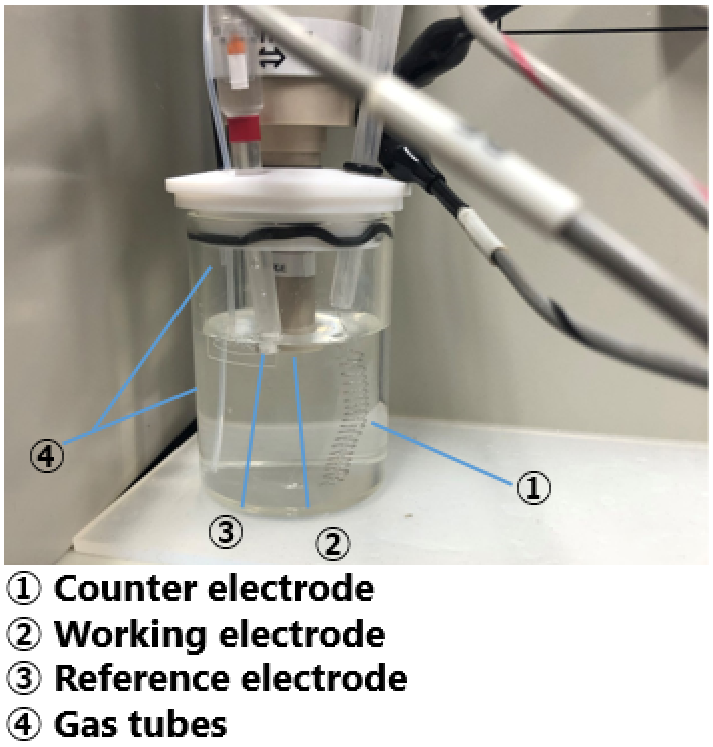

Fig. S2 The image of the electrochemical measurement system.

2. The comparison of the Cu-BTC pyrolyzed Cu@C by the XRD at various pyrolysis conditions

Table S1 The comparison of the Cu-BTC pyrolyzed Cu@C by the XRD at various pyrolysis conditions

Ref.	Pyrolysis conditions				Observed diffraction peaks in XRD	
	Temperature/°C	Time/h	Atmosphere	Pressure	Copper speices	Graphitic carbon
S1	350	2	N_2	1 atm	Cu, Cu_2O	No observed.
S1	350	2	H_2	1 atm	Cu, Cu_2O	No observed.
S2	400	1	5% H_2-N_2	1 atm	Cu	No observed.
S3	400	2	N_2	1 atm	Cu, Cu_2O*	Slightly observed?**
S4	500	-	N_2	1 atm	Cu, Cu_2O	No observed.
S5	400	1-4	N_2	1 atm	Cu, CuO, Cu_2O	No observed.
S5	500	1-4	N_2	1 atm	Cu, CuO, Cu_2O	No observed.
S5	600	1-4	N_2	1 atm	Cu, Cu_2O	No observed.
S5	700	1-4	N_2	1 atm	Cu, Cu_2O	No observed.
S6	700	4	Ar	1 atm	Cu, Cu_2O, CuO	Slightly observed after HCl washing.
S6	800	4	Ar	1 atm	Cu, Cu_2O, CuO	Slightly observed after HCl washing.
S7	800	2	Ar	1 atm	Cu	Observed after H_2SO_4 washing.
S8	850	8	Ar	1 atm	Cu, CuO	No observed.
**This work**	500	0.25	Vacuum-sealed	Vacuum	Cu, Cu_2O	No observed.
**This work**	500	0.25	Air	0.5 GPa	Cu, Cu_2O	No observed.
**This work**	500	0.25	Air	1 GPa	Cu, Cu_2O	No observed.
**This work**	500	0.25	Air	5 GPa	Cu	No observed.

*: the authors did not state the existence of the Cu_2O diffraction peaks in the XRD pattern, but the pattern had a peak at ~36° that seems to be corresponding to Cu_2O (111) plane. **: the authors state the peak of carbon was observed at 2θ=20°, but the pattern did not seem to have such a peak at the position.

3. TEM/STEM-EDS analysis of particles observed by STEM-HAADF

We conducted TEM/STEM-EDS measurements of Cu@C-5GPa, Cu@C-1GPa, and Cu@C-vac to confirm that those observed particles consist of copper. The point analysis of Cu@C-5GPa indicated a significant difference in the Cu peak intensity between the areas with and without particles (Fig. S3). The analysis of Cu@C-1GPa showed the same results (Fig. S4). For Cu@C-vac, the EDS mapping clearly shows that the observed particle consists of Cu (Fig. S5). Thus, it was confirmed that the observed particles in Fig. 2 were made of copper.

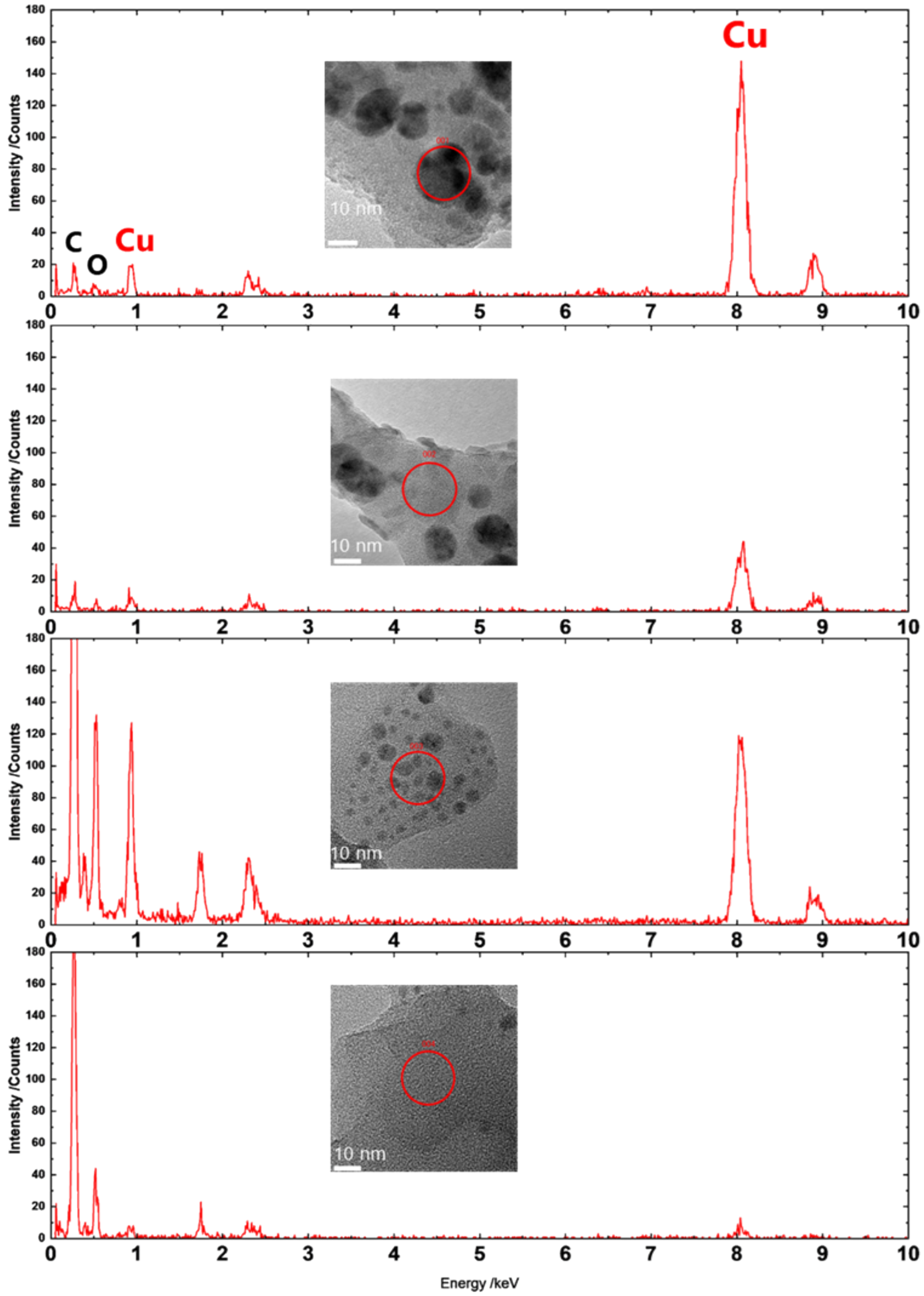

Fig. S3 the STEM-EDS elemental analysis at multiple points of the Cu@C-5GPa

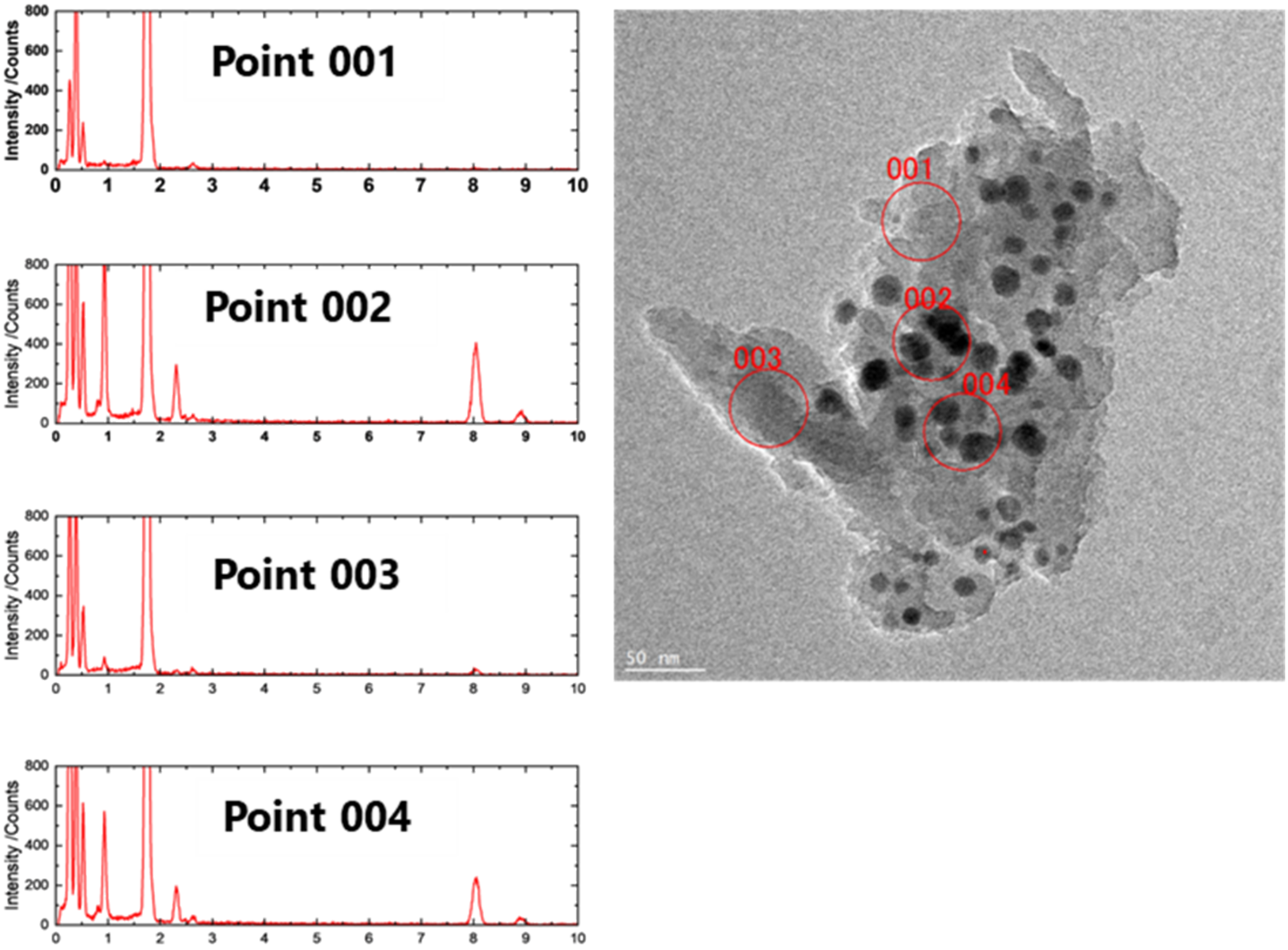

Fig. S4 the STEM-EDS elemental analysis at multiple points of the Cu@C-1GPa

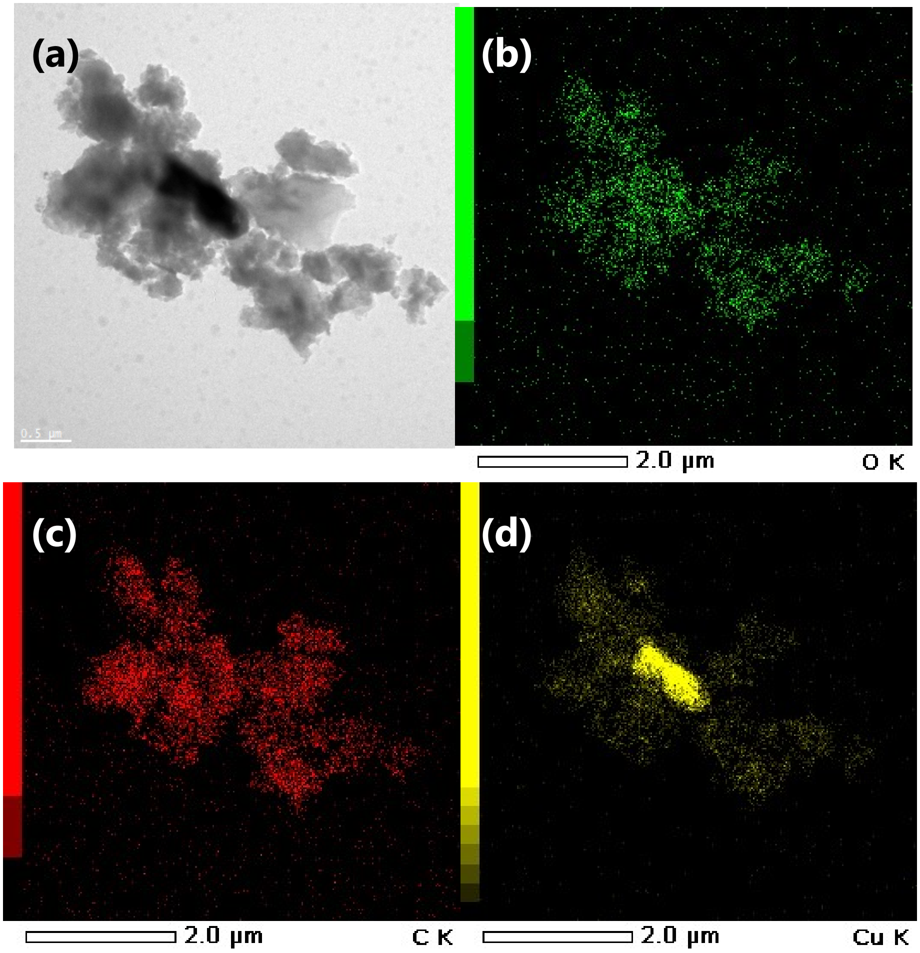

Fig. S5 (a)TEM image of Cu@C-vac and the STEM-EDS mapping images of (a): (b) C, (c) O, and (d) Cu.

4. XPS and AES for the analysis of Cu valency

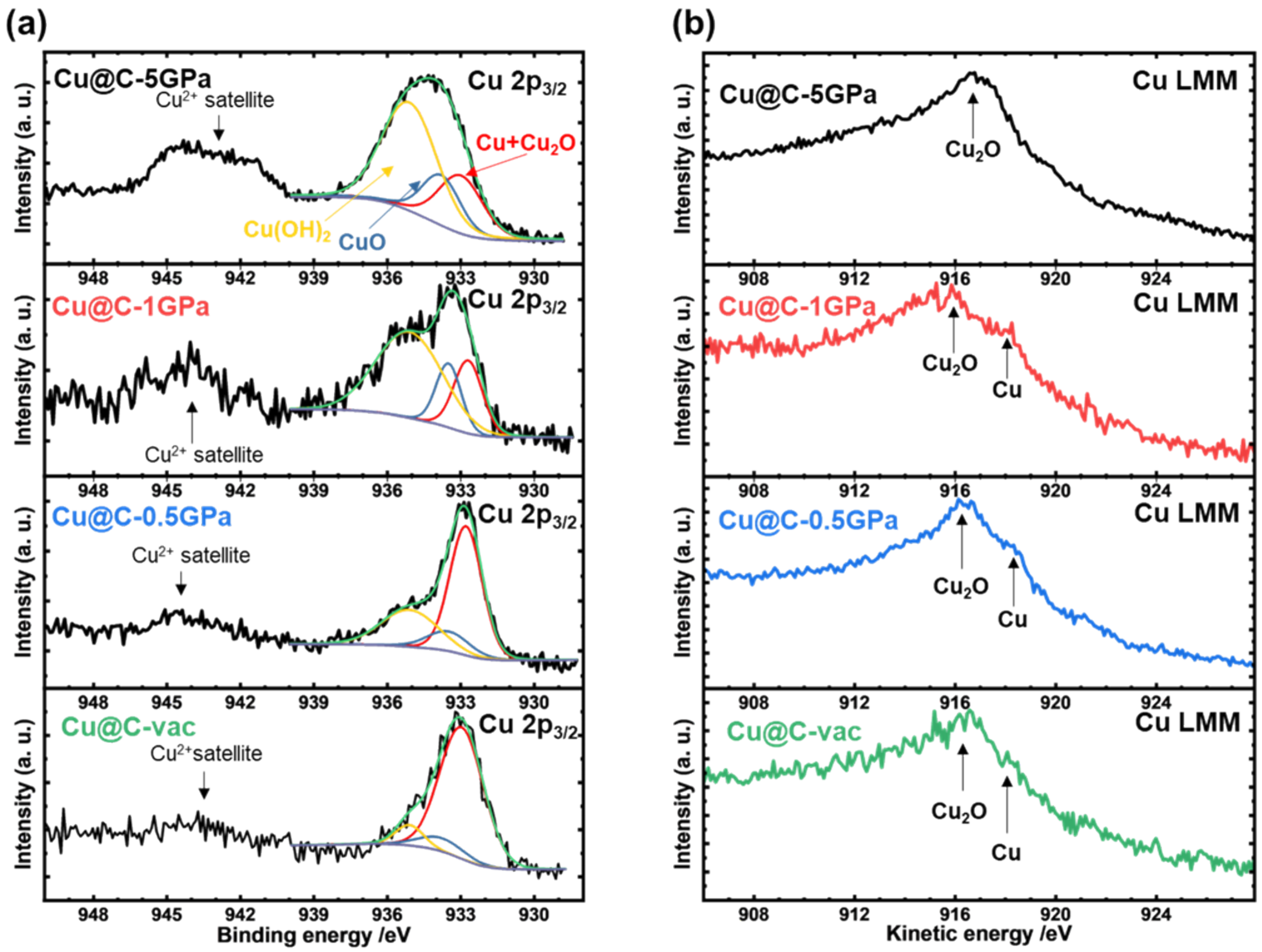

Fig. S6 (a) Cu $2p_{3/2}$ XPS spectra and curve fitting. Red, blue, yellow, green, and purple curve indicates Cu+Cu_2O component, CuO component, $Cu(OH)_2$ component, fitting curve, and background, respectively. (b)Cu LMM Auger spectra.

Table S2 The chemical composition of Cu calculated from Cu $2p_{3/2}$ XPS spectra.

Sample	composition		
	Cu+Cu_2O	CuO	$Cu(OH)_2$
**Cu@C-5GPa**	**24.3%**	**18.2%**	**57.5%**
**Cu@C-1GPa**	**22.4%**	**17.3%**	**60.3%**
Cu@C-0.5GPa	**58.6%**	**12.0%**	**29.4%**
**Cu@C-vac**	**83.0%**	**8.79%**	**8.26%**

5. Cyclic voltammetry of Cu@C-0.5GPa and literature

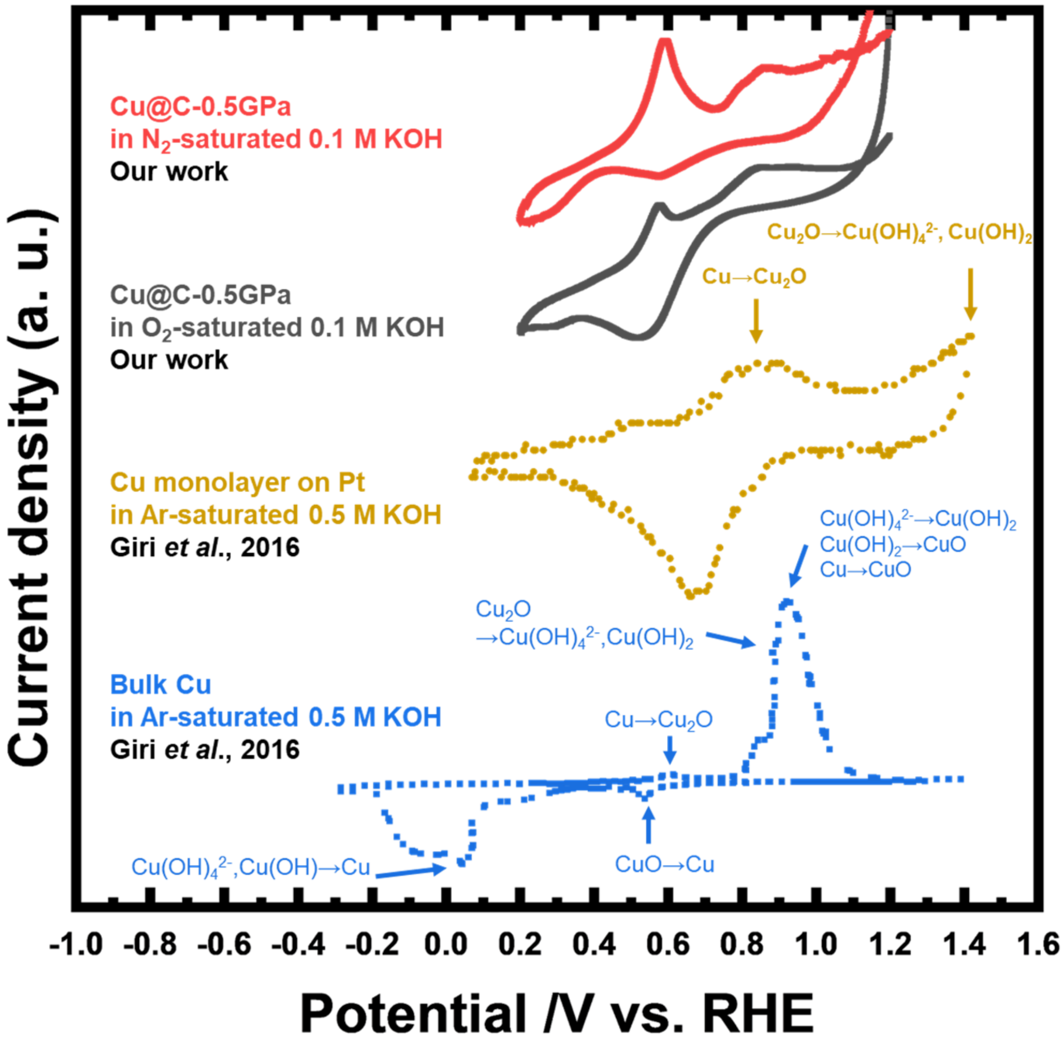

Fig. S7 The comparison of the CV curves of the Cu@C-0.5GPa in N_2/O_2-saturated 0.1 M KOH, that of the copper monolayer deposited on the 20 wt% platinum nanoparticles supported on carbon electrode in Ar-saturated 0.5 M KOH, and that of bulk copper electrode in Ar-saturated 0.5 M KOH. All curves were measured at scan rate 10 mV/s. Data from Cu monolayer and bulk Cu were digitized from figures in the works of ref. [9] and their potentials were converted from V vs. Hg/HgO to V vs. RHE by the following formula: $E_{RHE} = E_{Hg/HgO} + 0.098$ V + 0.0591×(pH of the 0.5 M KOH).

6. The comparison of the copper-based bifunctional electrocatalyst.

Table S3 The performance comparison of the reported copper-based bifunctional electrocatalyst. The values marked with * are data digitized from LSV curves in the works of literature. CNT stands for carbon nanotubes; NCNT stands for nitrogen-doped CNT; SAs stands for single-atoms; HNCNx stands for hollow nano-spheroids of nitrogen-deficient carbon nitride frameworks; NC stands for nitrogen-doped carbonaceous nanoleaves; NG stands for nitrogen-doped graphene; RGO stands for reduced graphene oxides.

Materials	Cu species Morphology	Medium	Tafel slope/mV dec^{-1}		Ref.
			ORR	OER	
(Cu, Co)$_3$OS$_3$ @CNT-C$_3$N$_4$	Nanoparticles @honeycomb-like porous nanosheets	0.1 M KOH	88	129	S10
20% Pt/C and 10% RuO$_2$	-	0.1 M KOH	89	230	S10
Ag$_{50}$Cu$_{50}$ @Cu	Nanoparticles @amorphous film	0.1 M KOH	109*	494*	S11
CuCo@CNT	Nanoparticles @nanotubes	0.1 M KOH	169*	280*	S12
Cu@NCNT	Nanoparticles @nanotubes	0.1 M KOH	112*	202*	S12
Cu@NCNT/Co$_x$O$_y$	Nanoparticles @nanotubes/nanoparticles	0.1 M KOH	106*	55(low *i*)* 145 (high *i*)*	S12
Cu-SAs@HNCNx	single atoms@ hollow nanospheroids	0.1 M KOH	43	64	S13
CuCo @NC	Nanoparticles @nanoleaves	0.1 M KOH(ORR), 1.0 M KOH(OER)	63	95	S14
AgCu alloy@Ni	Nanoparticles@foam	0.1 M KOH	83*	289*	S15
CuCo@NG	Nanoparticles@nanosheets	0.1 M KOH	58*	78	S16
Cu@NG	Nanoparticles@nanosheets	0.1 M KOH	51*	151	S16
CuNi@RGO	Nanoparticles@nanosheets	0.5M KOH	148*	182	S17
FeCu$_{0.3}$ @NCNT	Nanoparticles@nanotubes	0.1 M KOH	92.1	263.4	S18
**Cu@C-5GPa**	**Cluster@particles**	**0.1 M KOH**	**539 (low *i*) 115 (high *i*)**	**278**	**This work**
**Cu@C -1GPa**	**Nanoparticles@particles**	**0.1 M KOH**	**434 (low *i*) 92.7 (high *i*)**	**230**	**This work**
**Cu@C-0.5GPa**	**Thin shell@particles**	**0.1 M KOH**	**99.8**	**160**	**This work**
**Cu@C-vac**	**microparticles@particles**	**0.1 M KOH**	**424 (low *i*) 67.8 (high *i*)**	**244**	**This work**

References

S1 Y. Zhao, X. Wu, J. Zhou, Y. Wang, S. Wang, X. Ma, MOF-Derived Cu@C Catalyst for the Liquid-Phase Hydrogenation of Esters, *Chem. Lett.*, 2018, **47**, 883–886.

S2 Q. Gong, L.-P. Sun, Z. Wu, L.-H. Huo, H. Zhao, Enhanced Non-Enzymatic Glucose Sensing of Cu–BTC-Derived Porous Copper@carbon Agglomerate, *J. Mater. Sci.*, 2018, **53**, 7305–7315.

S3 H. Tan, C. Ma, L. Gao, Q. Li, Y. Song, F. Xu, T. Wang, L. Wang, Metal–Organic Framework-Derived Copper Nanoparticle@Carbon Nanocomposites as Peroxidase Mimics for Colorimetric Sensing of Ascorbic Acid, *Chem. Eur. J.*, 2014, **20**, 16377–16383.

S4 Y. Yao, X. Wu, O. Y. Gutiérrez, J. Ji, P. Jin, S. Wang, Y. Xu, Y. Zhao, S. Wang, X. Ma, J. A. Lercher, Roles of Cu+ and Cu0 Sites in Liquid-Phase Hydrogenation of Esters on Core-Shell CuZnx@C Catalysts, *Appl. Catal. B*, 2020, **267**, 118698.

S5 B. Wang, H. Pan, X. Lu, L. He, H. Zhang, X. Li, H. Guo, D. Zhou, Q. Xia, Copper-Organic Framework-Derived Porous Nanorods for Chemoselective Hydrogenation of Quinoline Compounds at an Aqueous/Oil Interface, *ACS Appl. Nano Mater.*, 2021, **4**, 11779–11790.

S6 I. A. Khan, A. Badshah, M. A. Nadeem, N. Haider, M. A. Nadeem, A Copper Based Metal-Organic Framework as Single Source for the Synthesis of Electrode Materials for High-Performance Supercapacitors and Glucose Sensing Applications, *Int. J. Hydrogen Energy*, 2014, **39**, 19609–19620.

S7 L. Qiao, A. Zhu, Y. Liu, Y. Bian, R. Dong, D. Zhong, H. Wu, J. Pan, Metal–Organic Framework-Driven Copper/Carbon Polyhedron: Synthesis, Characterization and the Role of Copper in Electrochemistry Properties, *J. Mater. Sci.*, 2018, **53**, 7755–7766.

S8 X. Zhang, J. Luo, P. Tang, J. R. Morante, J. Arbiol, C. Xu, Q. Li, J. Fransaer, Ultrasensitive Binder-Free Glucose Sensors Based on the Pyrolysis of in Situ Grown Cu MOF, *Sens. Actuators B Chem.*, 2018, **254**, 272–281.

S9 S. D. Giri, A. Sarkar, Electrochemical Study of Bulk and Monolayer Copper in Alkaline Solution, *J. Electrochem. Soc.*, 2016, **163**, H252.

S10 X. Wang, L. Peng, N. Xu, M. Wu, Y. Wang, J. Guo, S. Sun, J. Qiao, Cu/S-Occupation Bifunctional Oxygen Catalysts for Advanced Rechargeable Zinc–Air Batteries, *ACS Appl. Mater. Interfaces*, 2020, **12**, 52836–52844.

S11 X. Wu, F. Chen, Y. Jin, N. Zhang, R. L. Johnston, Silver–Copper Nanoalloy Catalyst Layer for Bifunctional Air Electrodes in Alkaline Media, *ACS Appl. Mater. Interfaces*, 2015, **7**, 17782–17791.

S12 X. Zhao, F. Li, R. Wang, J.-M. Seo, H.-J. Choi, S.-M. Jung, J. Mahmood, I.-Y. Jeon, J.-B. Baek, Controlled Fabrication of Hierarchically Structured Nitrogen-Doped Carbon Nanotubes as a Highly Active Bifunctional Oxygen Electrocatalyst, *Adv. Funct. Mater.*, 2017, **27**, 1605717.

S13 N. K. Wagh, S. S. Shinde, C. H. Lee, J.-Y. Jung, D.-H. Kim, S.-H. Kim, C. Lin, S. U. Lee, J.-H. Lee, Densely Colonized Isolated Cu-N Single Sites for Efficient Bifunctional Electrocatalysts and Rechargeable Advanced Zn-Air Batteries, *Appl. Catal. B*, 2020, **268**, 118746.

S14 M. Huo, B. Wang, C. Zhang, S. Ding, H. Yuan, Z. Liang, J. Qi, M. Chen, Y. Xu, W. Zhang, H. Zheng, R. Cao, 2D Metal–Organic Framework Derived CuCo Alloy Nanoparticles Encapsulated by Nitrogen-Doped Carbonaceous Nanoleaves for Efficient Bifunctional Oxygen Electrocatalyst and Zinc–Air Batteries, *Chem. Eur. J.*, 2019, **25**, 12780–

12788.

S15 Y. Jin, F. Chen, Facile Preparation of Ag-Cu Bifunctional Electrocatalysts for Zinc-Air Batteries, *Electrochim. Acta*, 2015, **158**, 437–445.

S16 P. Liu, Y. Hu, X. Liu, T. Wang, P. Xi, S. Xi, D. Gao, J. Wang, Cu and Co Nanoparticle-Co-Decorated N-Doped Graphene Nanosheets: A High Efficiency Bifunctional Electrocatalyst for Rechargeable Zn–Air Batteries, *J. Mater. Chem. A*, 2019, **7**, 12851–12858.

S17 A. M. Al-Enizi, M. Ubaidullah, J. Ahmed, H. Alrobei, S. M. Alshehri, Copper Nickel@reduced Graphene Oxide Nanocomposite as Bifunctional Electro-Catalyst for Excellent Oxygen Evolution and Oxygen Reduction Reactions, *Mater. Lett.*, 2020, **260**, 126969.

S18 B. Wang, L. Xu, G. Liu, Y. Ye, Y. Quan, C. Wang, W. Wei, W. Zhu, C. Xu, H. Li, J. Xia, In Situ Confinement Growth of Peasecod-like N-Doped Carbon Nanotubes Encapsulate Bimetallic FeCu Alloy as a Bifunctional Oxygen Reaction Cathode Electrocatalyst for Sustainable Energy Batteries, *J. Alloys Compd.*, 2020, **826**, 154152.
